# General public takes up counterintuitive expert advice on effective climate action

**DOI:** 10.1038/s41598-025-88122-0

**Published:** 2025-02-03

**Authors:** Johannes Jarke-Neuert, Grischa Perino, Daniela Flörchinger, Manuel Frondel

**Affiliations:** 1 Institute of Climate and Energy Systems—Jülich Systems Analysis (ICE-2), Forschungszentrum Jülich, Wilhelm-Johnen-Straße, 52428 Jülich, Germany; 2 Center for Earth System Research and Sustainability (CEN), Universität Hamburg, Bundesstraße 53, 20146 Hamburg, Germany; 3 Department of Socioeconomics, Universität Hamburg, Welckerstraße 8, 20354 Hamburg, Germany; 4 RWI—Leibniz Institute for Economic Research, Hohenzollernstraße 1-3, 45128 Essen, Germany; 5 Department of Economics, Ruhr-Universität Bochum, Universitätsstraße 150, 44801 Bochum, Germany

**Keywords:** Climate-change mitigation, Climate-change policy, Environmental economics, Psychology and behaviour

## Abstract

Individual voluntary climate action could contribute to closing the gap between global emission targets and the instruments in place. However, complex regulatory frameworks make it difficult for individuals to understand which actions align with their goals. Expert advice might provide guidance, but it is not trivial how detailed the advice should be. In a large consequential choice experiment with five informational load conditions, this paper uses the voluntary cancellation of European Union Allowances as an application to narrow down a minimum amount of information required to induce effective actions. We find a clear pattern of advice being processed and followed, even if it includes a demanding level of detail and counters prior convictions. Moreover, a mere assertion is highly effective already. These results are good news for efforts to increase the effectiveness of voluntary climate action.

## Introduction

There is an implementation gap in climate policy. The set of instruments in place is not sufficient to achieve the global and national emission targets.^1–5^ Individual voluntary climate action could help in closing this gap. Many people worldwide are willing to act,^6,7^ but often do not for a variety of reasons that can be broadly classified into “structural” and “psychological barriers”.^8–10^ At the intersection of the two classes is *complex overlaps*: the effect of individual action depends on the details of the prevailing public policy framework in a way that is difficult to grasp and sometimes highly counter-intuitive.^11^ For example, replacing an old, inefficient refrigerator can actually harm the climate if the electricity supplier is subject to a cap-and-trade system.^12^ As a result, knowing which actions are most effective in reducing emissions and making trade-offs between different actions is challenging.^13^ The problem may be amplified by the fact that the topic of climate change is emotionally charged,^14–17^ which might interfere with individuals’ willingness or ability to process all available information in an unbiased way.

Expert advice might be an effective instrument to guide individual decisions. However, it is yet unclear how detailed this advice should be to trigger the intended behavioral response: an assertion alone—even if objectively correct—may not be persuasive, as it contradicts prior beliefs or dearly-held convictions. Notably, due to two psychological pitfalls, advisors could fail to convince people of the recommended option: First, Simplicity Theory formalizes the well-documented notion that people tend to be averse to complexity, and hence may prefer inferior but simple options over superior alternatives that are difficult to process.^18–20^ As a result, people may ignore the advice and instead follow their intuition or prior beliefs,^21–23^ or withdraw from the decision situation altogether. Second, Reactance Theory holds that people dislike interference—such as attempts of persuasion—with their behavioral freedom.^24,25^ If an advice is perceived as offensive in that way, people tend to explicitly not follow or even do the exact opposite. The perception may be amplified by lengthy explanations.

Hence, there may be a delicate trade-off to be solved when advising people about the most effective way of individual abatement action: the information provided must be persuasive, yet minimal enough to not run into the above pitfalls. In the present paper, we address two questions: What is the minimum amount of information required to induce participants to choose a more effective carbon abatement action? Is there evidence that informational load exceeding this point triggers complexity aversion or reactance in relevant magnitude?

We take the EU Emission Trading System (ETS) as a starting point, but our results translate to other settings in which complex underlying conditions may impede individuals from making a choice that best adheres to what they intend to achieve. Cancellation of EUAs in the EU ETS is a viable voluntary abatement option for private households and firms offered by several commercial and non-profit organizations. German providers have so far set aside more than 120,000 EUAs for later cancellations based on donations from private parties. At current market prices, they have a value of about €10 million. Moreover, the EU encourages member states to supplement mandatory coal phase-outs with cancellations of EUAs and provides a legal framework for doing so (Art. 12(4) of Directive 2003/87/EC).

Up to 2018, the cancellation of one EUA was equivalent to saving one ton of carbon dioxide. This simple one-to-one correspondence ended with the introduction of the so-called Market Stability Reserve (MSR). Since this ETS reform, the supply of EUAs is dynamically adjusted pending on the number of EUAs in circulation, and the reduction of emissions depends on the *timing* of cancellation,^26,27^ immediate canceling being less effective than delaying it by one year or more.

However, the time dependence of cancellations is not known beyond a small circle of experts. Moreover, the underlying mechanisms are difficult to convey to non-experts and require a fair amount of attention and cognitive effort. Finally, delay of cancellation is most likely counter-intuitive for most people, as it conflicts with the widespread belief that immediate action is needed to solve the climate crisis.^7^ Even the cancellation provision embedded in the legal framework of the EU ETS under which member states have canceled about 668,000 EUAs up to December 2023 only allows for immediate cancellations which is strictly dominated by delayed cancellation.^28^

There is substantial confusion about the effectiveness of cancellations as well as other supplementary measures in the context of the EU ETS in the private sector and among governments. For example, the London-based fintech SparkChange states that it has collected US$ 140 million to reduce emissions by merely holding and later selling EUAs, i.e. without actual cancellations. It claims to reduce total emissions to an extent that according to the scientific literature could only be realized by delayed cancellations.^29^ The latter are offered by a competitor, CAP2 GmbH.^30^ Hence, deeply conflicting claims are used to market carbon offsets via the EU ETS.

More broadly, the effectiveness of additional abatement efforts in the sectors covered by the EU ETS is driven by the very same mechanisms that render the timing of cancellations important.^26^ Conflicting beliefs about the climate benefit of such measures have contributed to the failure of the German coalition government in November 2024.^31^

We address the effectiveness of expert advice on the effectiveness of individual climate action in the context of the EU ETS in a large consequential choice experiment with subjects randomly distributed to five conditions with escalating informational load. All conditions involved the decision between (i) cancellation of an EUA and (ii) a €5 worth *Amazon* voucher.^32–35^ The “no timing” condition $z=1$ consisted of just this dichotomous choice. The “simple timing & zero advice” condition $z=2$ was identical to the first, except that it also involved the decision of whether the EUA should be canceled immediately or one year after the experiment. The “simple timing & minimal advice” condition $z=3$ and the “simple timing & extensive advice” condition $z=4$ were identical to $z=2$, except that participants were provided with explanations of the delayed EUA cancellation option being more effective prior to their decision. Condition $z=3$ only added a short sentence, whereas condition $z=4$ entailed a very detailed explanation. The level of detail makes the explanation more credible, but increases informational load significantly. Finally, the explanation in the “sophisticated timing & extensive advice” condition $z=5$ had the same level of detail as in condition $z=4$, but the delay was not one year but an unspecified period, weakly larger than one year, such that the abatement impact of the cancellation is maximized. This add-on condition reflects the offer made by several agencies in the real world, such as ForTomorrow gGmbH, Compensators e.V. and the Climate Concept Foundation, and it involves an even higher cognitive load than $z=4$, as it requires filling the ambiguity with some form of belief. All advices and explanations come from one of the authors of this study, who is a distinguished expert on the matter.

## Methods

A random sample of 4444 subjects was drawn from the spring 2021 wave of the German Socio-Ecological Panel,^36^ collected in collaboration with the survey institute *forsa*. The full wave surveyed 8677 individuals online. The sample is a broad cross-section of the German population, but is not representative (Suppl. Tab. 9). Participation was voluntary. 146 subjects dropped out before the experiment, 159 during the experiment, leaving us with a final sample of 4139 participants. The experiment was implemented via questionnaire, which was subject to cognitive pretesting.^37^

We designed four items to measure motivational aspects before the experimental part of the survey. Responses to all items were measured on a five-point ordinal scale and ranged from “fully disagree” to “fully agree”. For all items we constructed binary variables that take on the value 1 when the respondent agreed or fully agreed with the statement and 0 otherwise. The first item asks for the degree of agreement with the proposition “It is my moral duty to make an active contribution to climate protection.” (“Moral Duty”). Disagreement with the second statement “Effective climate protection can only be done by public policy.” (“Public policy”) measures the belief that personal action can make a difference. A temporal dimension is added with the statement: “The climate problem will not tolerate any delay. We must act now.” (“Urgency”). This item is designed to measure the belief that timing matters in climate action. The fourth item serves to separate out the opposite end of the motivational spectrum, asking for the degree of agreement with the statement “The climate issue is overrated.”.

The main experiment followed. First, a general introductory text that explained the basic decision task was shown. The lottery method was used to economize on study costs: one out of four randomly drawn choices was actually implemented, and participants knew. A second text then explained in simple but accurate terms the procedure of carbon abatement by canceling an EUA. Those texts were shown to all participants. Yet, no further details about how the EUA cancellation translates into climate protection were given at this point. Such details were the locus of experimental manipulation.

Subjects were randomly assigned to one out of the five experimental conditions. Across conditions, the questionnaire was purposefully varied slightly so that differences in response behavior can be causally attributed to the questionnaire variations. The variation was limited to one out of the ten questions that were used for the present study. The result of the condition assignment process is summarized in Suppl. Tab. 7.

In all conditions, the vouchers were sent out and the EUAs were purchased shortly after the study. The only difference between $z=2$ vs. $z=1$ was that in addition to choosing between cancellation (at an unspecified point in time) and the voucher, in $z=2$ they could choose between two specific timings of the cancellation (2021 and 2022) and the voucher. Condition $z=2$ provided no information on the implications of the timing of EUA cancellation. Condition $z=3$ added a short sentence stating that immediate cancellation reduces carbon emissions by 0.24 tons less than retirement one year after the experiment. Condition $z=4$ entailed a very detailed explanation of why the relative effectiveness of the two abatement options is as stated, with reference to the MSR. Finally, the explanation in condition $z=5$ had the same level of detail as in $z=4$, but the delay was not one year but an unspecified period, weakly larger than one year, such that the abatement impact of the cancellation is maximized. Following survey research standards, there was also a non-response option on choice screens. The full transcript of the instructions and questionnaires is provided in Suppl. Sec. E.

The outcomes of interest for this study are the willingness to contribute to climate change mitigation and the relative effectiveness of those contributions. For the first outcome, we define $a=1$ to indicate the choice of EUA retirement, whereas $a=0$ absorbs the two alternatives, the Amazon voucher and the no-choice alternative, and we term the probability $\Pr \left( a=1\right)$ “willingness to contribute” (WTC). We have deliberately set the value of the voucher much lower than the EUA market price as it is just an instrument to avoid pollution of the data by participants that do not care about the task, and to have EUA retirement rates high in the control conditions to leave enough scope for treatment effects (which were expected to be negative). That said, we are not interested in the absolute value of WTC but the differences between experimental conditions. For the second outcome, we define $d=1$ to indicate the choice of delayed EUA retirement, where the reference category $d=0$ is immediate retirement, and we term the probability $\Pr \left( d=1\right)$ “relative effectiveness of contribution” (REC).

To probe for the robustness of results we construct binary variables indicating whether subjects paid attention to the experiment using the time spent on the introduction and advice screens. Suppl. Tab. 4 summarizes the median duration spent on each screen. Since the information presented on each screen is extensive, it is unlikely that respondents who spent less than the median duration on a given screen have actually read the information. We define a dummy variable that is equal to 1 if respondents spent at least the median time on the introductory screen, and 0 otherwise. Only treatment groups $z = 4,5$ received separate screens with advice. To construct a variable indicating whether respondents paid attention to the explanations provided for all survey participants, we sum up time spent on the introduction and the advice screens and define the dummy variable that is equal to 1 if this duration was greater of equal than the median in the respondent’s treatment group.

We measured need for cognition post-experimentally by means of the standard Need for Cognition (NFC) test.^38,39^ The test consists of four statements to which the respondent can express applicability on a seven-point Likert scale (1 = does not apply at all, 7 = does fully apply): (i) “It is enough for me simply to know the answer without understanding the reasons for the answer of a problem.”; (ii) “I like my life to be full of tricky tasks to solve.”; (iii) “I would prefer more complicated problems to simple problems.”; and (iv) “First and foremost, I think because I have to.”. From the four items we constructed a simple need for cognition score for each participant by summing up the response codes (appropriately inverting the scales for the first and last item). Thus, the minimum achievable score is 4 and the maximum is 28. We classify individuals with a score at or below the mid-point as having a low need for cognition and those with a score above the mid-point as having a high need for cognition. We opt for this conservative classification because we have no reason to assume that the NFC measure is actually interval-scaled.

To analyze the effects of treatment assignment on WTC and REC, we estimated maximum likelihood probit regressions with bootstrap standard errors of the respective two indicators onto the treatment indicators and a vector of covariates. If randomization of treatment assignment is successful, including covariates is not necessary for identifying treatment effects. Nevertheless, we conduct additional analyses with covariates to assess the robustness of our results (Suppl. Tab. 12 and 14).

To estimate heterogeneous treatment effects, we extend the regression equation by including the respective variable of interest and its interaction with treatment assignment. When investigating the moderating effects of motivated reasoning, we include all four motivational variables and their interaction with treatment assignment within the same estimation.

The study was pre-registered in the Randomized Controlled Trial Registry of the American Economic Association under code AEARCTR-0007372. Research Ethics Board approval has been granted by the Vice-Dean for Research of the Faculty of Economics and Social Sciences of the Universität Hamburg, Prof. Alexander Szimayer, on April 30, 2021. All methods were carried out in accordance with relevant guidelines and regulations. Informed consent was obtained from all subjects.

## Results

### Willingness to contribute with non-explained timing lower than without timing, but not different when timing is explained

**Figure 1:**
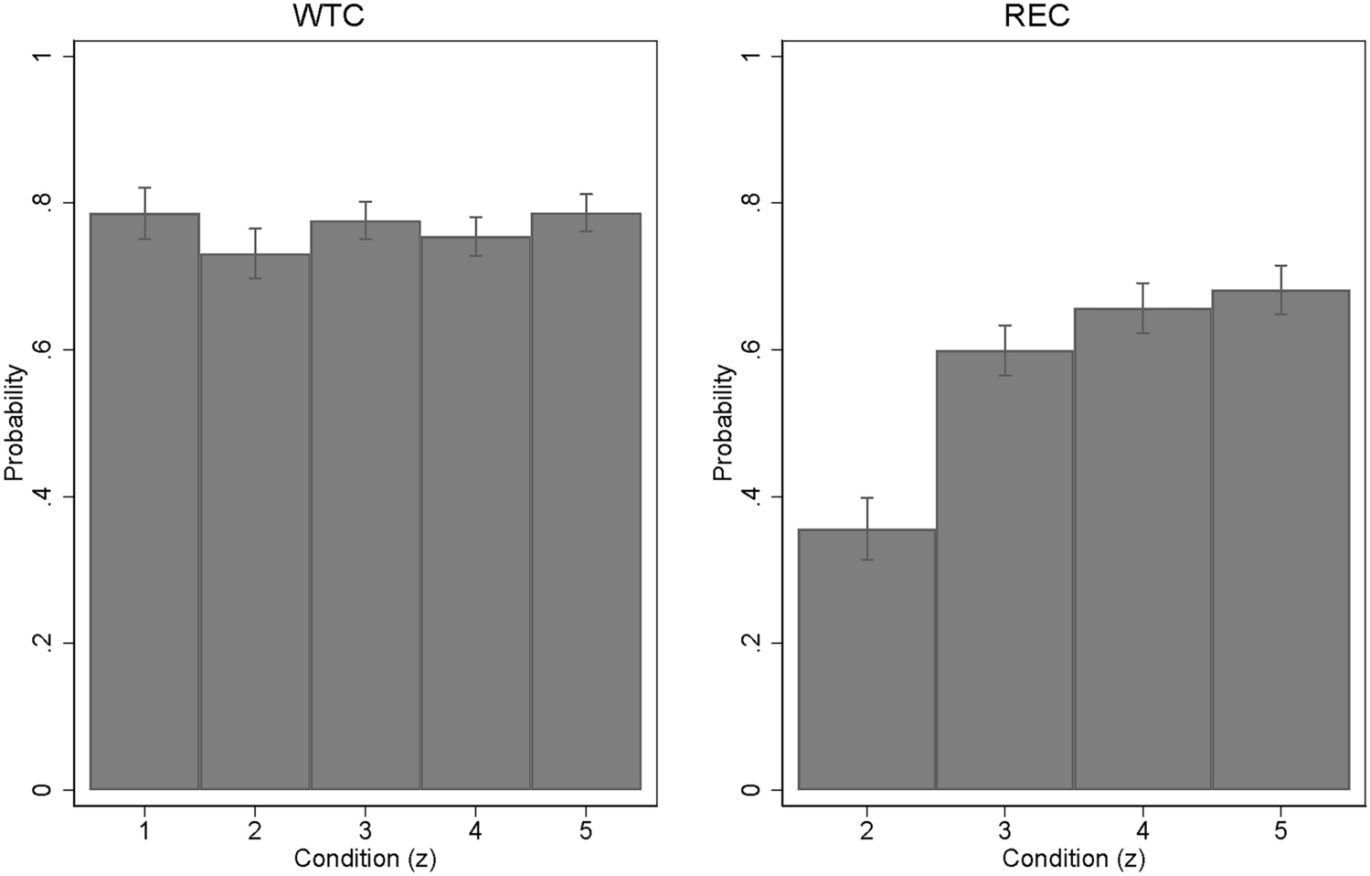
WTC (left-hand) and REC (right-hand). Margins and 95%-confidence intervals from maximum likelihood probit estimation of $\Pr \left( a=1\mid z\right)$ and $\Pr \left( d=1\mid a=1,z\right)$. Full estimation results in Tables 10 and 14, supplementary information file. The number of observations is 4139 for WTC and 2790 for REC.

Condition $z=1$ sets the baseline of WTC: of the 491 subjects in that condition, 386 subjects have chosen to cancel an EUA, 68 subjects selected the voucher, and 37 subjects the non-response category, yielding $\Pr \left( a=1\mid z=1\right) =78.6 \%$ (Fig. 1).

**Figure 2:**
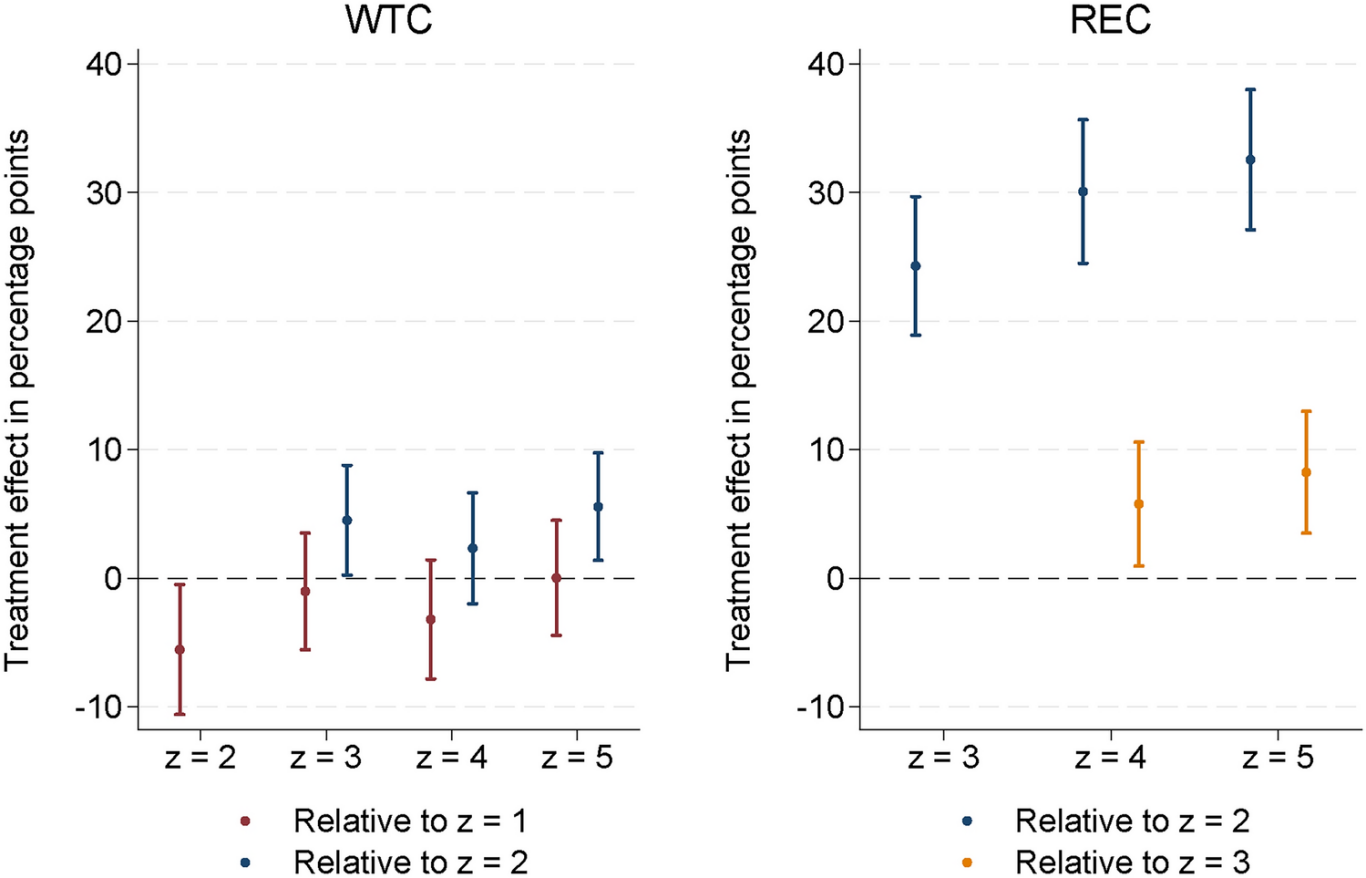
Average treatment effects on WTC (left-hand) and REC (right-hand). Average marginal effects and 95%-confidence intervals from maximum likelihood probit estimation of $\Pr \left( a=1\mid z\right)$ and $\Pr \left( d=1\mid a=1,z\right)$. Full estimation results in Tab. 10, 11, 14, and 15, supplementary information file. The number of observations is 4139 for treatment effects on WTC relative to $z=1$ and 3648 for effects relative to $z=2$; 2790 for treatment effects on REC relative to $z=2$ and 2304 for effects relative to $z=3$.

WTC differs between the “no timing” ($z=1$) and the “simple timing & zero advice” condition ($z=2$), albeit the size of the effect $\Pr \left( a=1\mid z=2\right) -\Pr \left( a=1\mid z=1\right)$ of $-5.5$ percentage points is small relative to the reference probability (Fig. 2, in red). Thus, adding a temporal dimension to the decision is relevant, but not perceived as a big change in the decision context. Given that respondents have a choice between early and delayed retirement, providing information on the relative effectiveness of these options can increase overall WTC (Fig. 2, in blue), although the effects are small, ranging from 2.3 to 5.6 percentage points, and not statistically significant for the extensive explanation in $z=4$.

We do not find strong evidence of complexity aversion or reactance in the treatment effects on the WTC. Point estimates range from $-1$ and $-3$ percentage points in the advice conditions relative to the baseline $z=1$, and none is statistically significantly different from zero (Fig. 2, in red). WTC is also approximately the same in the simple and extensive advice conditions. Sensitivity analyses are available in Suppl. Tab. 12 and 13.

### Contributions are more effective with advice vs. no advice

The “simple timing & zero advice” condition ($z=2$) is the baseline for the REC metric. Under this condition—where the delayed cancellation option was not further explained—there is reason to expect that immediate EUA cancellation is the intuitive response for most subjects (Intro.). They may also get a sensation of “warm glow” from immediate cancellation.^40^ This suggests that we should observe $\Pr \left( d=1\mid a=1,z=2\right) <0.5$. This is what happened: of the 486 subjects in condition $z=2$ who have chosen to cancel an EUA, 313 selected immediate EUA retirement and 173 delayed retirement, yielding $\Pr \left( d=1\mid a=1,z=2\right) = 35.6\%$ (Fig. 1).

In conditions $z>2$ the instructions contain the advice that delayed cancellation saves more emissions than immediate cancellation, albeit in different degrees of detail. Specifically, the advice in the “simple timing & minimal advice” condition ($z=3$) is little more than an assertion. The hazard that such minimal advice may not be persuasive would materialize in REC being not significantly higher than in the “simple timing & zero advice” condition, $\Pr \left( d=1\mid a=1,z=3\right) \lessapprox \Pr \left( d=1\mid a=1,z=2\right)$. Otherwise, for those who are willing to contribute, the information provided reveals a strict dominance in effectiveness at identical opportunity costs, shifting probability mass towards late cancellation. Indeed, we observe that even the minimal advice increases REC significantly by 24.3 percentage points relative to zero advice (Fig. 2, in blue).

Furthermore, we do not find traces of complexity aversion or reactance in the treatment effects on REC. In the extensive advice conditions, REC is also significantly higher compared to zero advice (Fig. 2, in blue), and even significantly larger than in the minimal advice condition (by 5.8 percentage points in $z=4$ and by 8.3 percentage points in $z=5$; Fig. 2, in orange). The estimates are almost the same when the regression controls for gender and income (Suppl. Table 14), the two socio-economic variables for which we found statistically significant differences between the treatment groups despite randomization (Suppl. Tab. 8). Thus, additional informational load in fact enhances REC, albeit the incremental effect is decreasing and the by far greatest part of the respective overall effects can be attributed to the minimal assertion that delayed cancellation is more abatement-effective.

### No significant heterogeneity of treatment effects with respect to normative motivations and need for cognition

We conclude the analysis with a note on treatment effect heterogeneity. Specifically, three aspects are likely to moderate the individual information processing and decision-making process in the EUA cancellation task: attention paid to the information provided, motivated reasoning, and need for cognition.

**Figure 3:**
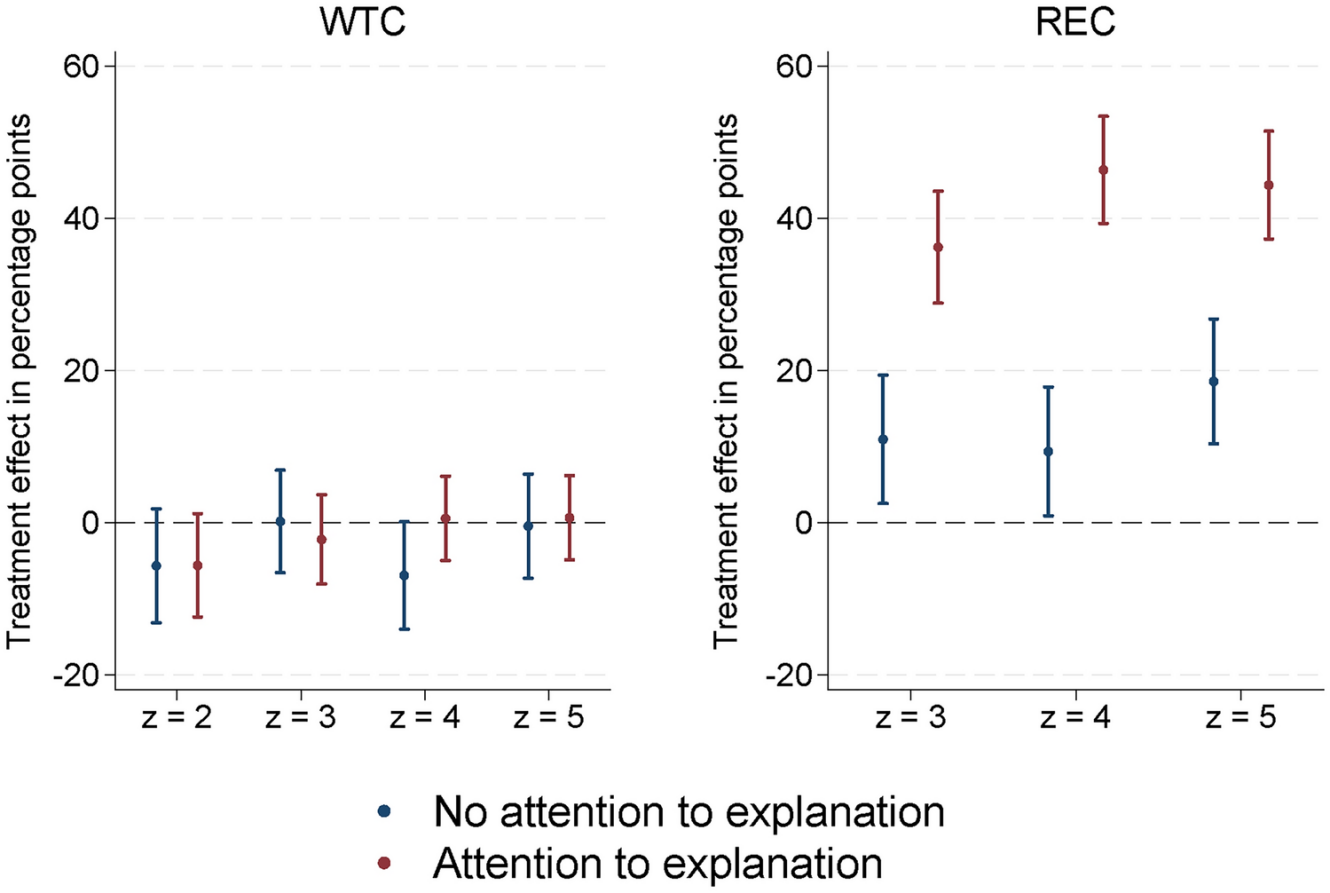
Heterogeneous treatment effects by attention paid to the introduction and explanations. Average marginal effects and 95%-confidence intervals from maximum likelihood probit estimation of $\Pr \left( a=1\mid z\right)$ and $\Pr \left( d=1\mid a=1,z\right)$. Full estimation results in Tab. 16, supplementary information file. The number of observations is 4135 for WTC and 2788 for REC.

The effect of expert advice is likely to depend on how much attention individuals pay to it. Therefore, we investigate treatment effect heterogeneity by whether respondents pay attention to the information provided on the introductory and advice screens (Fig. 3). For WTC, treatment effects barely differ between attentive and inattentive individuals. For REC, by contrast, the treatments are much more effective among respondents who pay more attention, by 25 to 37 percentage points. This makes sense, because the information provided concerns the relative effectiveness of the two mitigation options. However, it is reassuring that there is still a positive treatment effect of 11 to 19 percentage points among respondents who are less willing to engage with complex and counter-intuitive information. Since attention paid to the advice screens might already be influenced by the treatments, we redo the analyses using only attention to the introduction screens in Suppl. Tab. 17. Results barely change.

**Figure 4:**
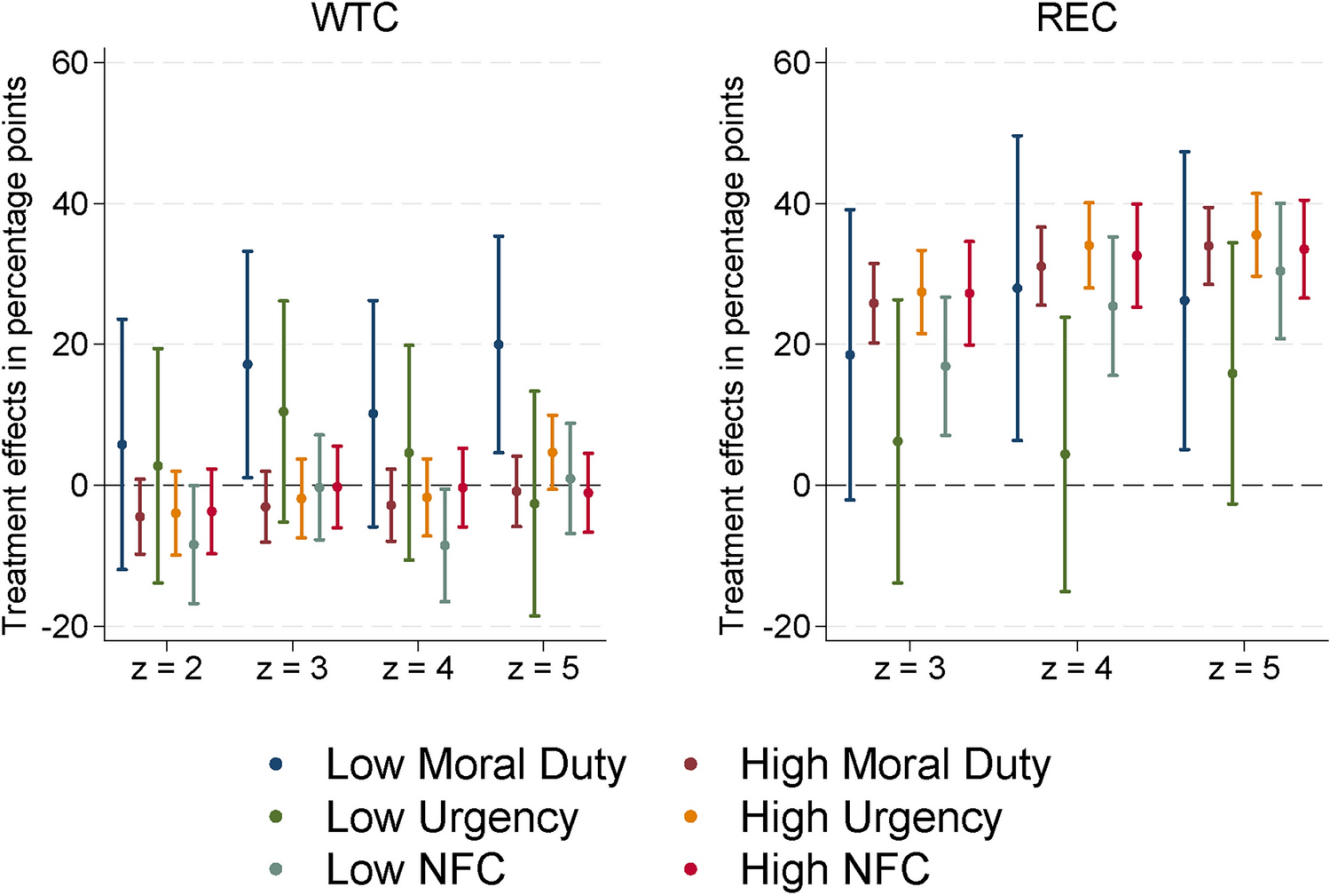
Heterogeneous treatment effects by motivational aspects and need for cognition (NFC). Average marginal effects and 95%-confidence intervals from maximum likelihood probit estimation of $\Pr \left( a=1\mid z\right)$ and $\Pr \left( d=1\mid a=1,z\right)$. Full estimation results in Tables 5 and 6, supplementary information file.

Motivated beliefs are difficult to update, as they tend to be more inert to conflicting information than “rational” beliefs.^41–44^ Hence, the treatment effects could be weaker in individuals that have strong normative beliefs regarding the individual moral duty to act and who believe that action is urgent (compared with individuals with more moderate beliefs), as the advice is less likely to change those beliefs. We measured such normative beliefs using a pre-experimental item battery in the questionnaire (Suppl. Sec. A) and extended the probit regressions with interaction terms: there is no strong evidence of treatment effect heterogeneity with respect to motivated beliefs (Fig. 4). In fact, the treatment effects on REC tend to be a bit larger in highly motivated subjects, which is the opposite of what motivated reasoning theory would suggest. We also use the item battery to probe for a potential experimenter demand effect: the pull of an experimenter demand effect over and above the informational value of the advice should be independent of any attitude towards the issue at hand (Suppl. Sec. C); hence, given the heterogeneity in beliefs for urgent action, those who think the need for climate action is less pressing should delay retirement more often. Yet, we find the opposite.

Another possible source of treatment effect heterogeneity follows from the fact that understanding the experimental instructions—and especially the extensive advice in conditions $z=4$ and $z=5$—requires cognitive effort in the form of information processing. People generally tend to avoid cognitive load, but individuals differ.^45^ We measured this heterogeneity post-experimentally by means of the Need for Cognition (NFC) test,^38,39^ and again extended the probit regressions with interaction terms (Fig. 4): it turns out that there is no evidence of treatment effect heterogeneity with respect to NFC.

## Discussion

One potential avenue for reducing the implementation gap in climate policy is to increase the effectiveness of voluntary climate action.^2–4^ In many contexts, though, individuals face substantial complexity in the form of regulatory environments and market interactions. Then, expert knowledge is required to identify the most effective choice for climate action. However, it is not straightforward how extensive expert advice would need to be to trigger behavioral change in individuals. If it is too short, it may not be convincing, whereas lengthy explanations might generate backlash.

The results of this study are good news for communicators aiming to increase the effectiveness of voluntary abatement. First, individuals display an apparent willingness to engage with complex frameworks. But even if they are reluctant to do so, expert advice can improve outcomes, albeit to a smaller extent. Second, minimal advice is sufficient to yield a sizable improvement in effectiveness. Third, more extensive explanations do not generate backlash, but lead to even better outcomes. Finally, although climate change is a very emotionally and ideologically charged topic, the uptake of information provided was most pronounced by individuals who most strongly believed in the opposite ranking, which is consistent with rational belief updating, but not with motivated reasoning.

There are caveats. First, our sample of subjects is slightly more educated than the overall German population (Suppl. Tab. 9), such that the results may be different in a population with lower education. Second, randomization of treatments may have not worked perfectly in our experiment, as the sample assigned to the “simple timing & zero advice” condition $z=2$ stands out slightly in terms of gender and income distributions (Suppl. Tab. 8). Since that condition is also an important baseline in our study, this is unfortunate in principle, as the treatment effects may be biased. However, when controlling for these co-variates statistically via regression analysis, the treatment effects stand robust (Suppl. Tab. 12 and 14). There are also caveats regarding the conclusions from the results and their application in the real-world. First, individuals might not be able to distinguish between true and false expert messages, such that misleading advice may exacerbate ineffective outcomes. Second, as noted above, lack of information or processing generally accounts only partially for the problem of inaction, other “barriers” may dominate in particular cases. Specifically, the real opportunity cost of action may be substantially greater than the €5 in our experiment. However, while the opportunity costs of engaging in climate action *at all* was €5, the opportunity cost of *choosing between the effective and the ineffective measure* was limited to the acquisition of information. In the real world the opportunity cost of being more effective might be both positive or negative. The latter in particular if one looks at cost per unit of impact.

In principle, the problem of complex regulatory frameworks inducing individual inaction may extend beyond the setting we are studying. For example, farmers seeking EU funding or subsidies could be deterred by complicated rules or conditions. This appears to be a fruitful avenue for further research.

## Supplementary Information

Supplementary Information.

## Data Availability

All data and materials of the study are freely available online at the Open Science Framework (OSF) under DOI 10.17605/OSF.IO/5HUCM.
