# Supplementary material for: General public takes up counterintuitive expert advice on effective climate action
Source: Sci Rep. 2025 Feb 3;15:4136. doi: 10.1038/s41598-025-88122-0 (PMC11790887; doi:10.1038/s41598-025-88122-0)
Supplement: Supplementary file 1 — Supplementary Information. [file 41598_2025_88122_MOESM1_ESM.pdf]

Supplementary Information for “General public takes up counterintuitive expert advice on effective climate action”

Johannes Jarke-Neuert[1,2,*], Grischa Perino[2,3], Daniela Flörchinger[4], and Manuel Frondel[4,5]

[1]Institute of Climate and Energy Systems—Jülich Systems Analysis (ICE-2) at Forschungszentrum Jülich, Wilhelm-Johnen-Straße, 52428 Jülich, Germany.
[2]Center of Earth System Research and Sustainability (CEN) at Universität Hamburg, Bundesstraße 53, 20146 Hamburg, Germany.
[3]Department of Socioeconomics at Universität Hamburg, Von-Melle-Park 9, 20146 Hamburg, Germany.
[4]RWI—Leibniz Institute for Economic Research, Hohenzollernstraße 1-3, 45128 Essen, Germany.
[5]Department of Economics, Ruhr-Universität Bochum, Universitätsstraße 150, 44801 Bochum, Germany.
[*]j.jarke-neuert@fz-juelich.de

ABSTRACT

This document contains a more detailed description of the information elictited pre-experimentally from subjects (Sec. A), a description of experimental manipulation checks (Sec. B), additional analysis of treatment effect heterogeneity (Sec. C), supplementary tables (Sec. D), and a full transscript of the experimental instructions presented to subjects (Sec. E).

A Pre-Experimental Items

The questionnaire started with a battery of five pre-experimental items that were presented to all participants and served to measure preferences, beliefs, and knowledge with respect to climate change and mitigation policy. At this point, the responses provide insights about relevant priors in the population.

A.1 Motivational Aspects

To measure motivational aspects, four items were designed, the first of which asks for the degree of agreement with the proposition “It is my moral duty to make an active contribution to climate protection.”. Responses to all items were measured on a five-point ordinal scale and ranged from “fully disagree” to “fully agree” (Tab. 1). 82.0 % of the respondents agree or fully agree with this statement – see the row on “Moral duty”. This result is taken as evidence that a vast majority of participants feels intrinsically urged to make a personal contribution to climate action.

Supplementary Table 1. Responses to four pre-experimental items measuring motivational aspects.

Item	fully disagree	rather disagree	neither nor	rather agree	fully agree	n/a[e]
Moral duty[a]	175	208	348	1,837	1,554	17
	4.2%	5.0%	8.4%	44.4%	37.6%	0.4%
Public policy[b]	347	887	785	1,500	595	25
	8.4%	21.4%	19.0%	36.2%	14.4%	0.6%
Urgency[c]	196	261	359	1,363	1,940	20
	4.7%	6.3%	8.7%	32.9%	46.9%	0.5%
Hype[d]	1,890	1,022	446	498	268	15
	45.7%	24.7%	10.8%	12.0%	6.5%	0.4%

[a] “It is my moral duty to make an active contribution to climate protection.”
[b] “Effective climate protection can only be done by public policy.”
[c] “The climate problem will not tolerate any delay. We must act now.”
[d] “The climate issue is overrated.”.
[e] Non-response category “don’t know/prefer not to say”

The second row of Tab. 1 lists the responses to the statement “Effective climate protection can only be done by public policy” (“Public policy”). Disagreement with this statement is considered to measure the belief that personal action can make a

difference. While there is some heterogeneity, the absolute majority of respondents believes that effective climate protection can only be achieved by public policy; in other words, personal action does not have a significant impact.

A temporal dimension is added with the statement: “The climate problem will not tolerate any delay. We must act now” (“Urgency”). This item is designed to measure the belief that timing matters in climate action, with a preference for immediate over delayed actions. The vast majority of about 80 % agrees or fully agrees with this statement (Tab. 1).

The fourth item serves to separate out the opposite end of the motivational spectrum, asking for the degree of agreement with the statement “The climate issue is overrated.”. Responses are shown in the fourth row of Tab. 1 (“Hype”). A majority of about 70 % disagrees or fully disagrees, but there is a non-negligible minority of about 19 % of respondents who agree or fully agree. These individuals can be expected to be weakly or not at all motivated to make a personal contribution to climate action, nor to spend significant cognitive effort to engage with climate policy instruments.

While these four items refer to clearly distinct aspects of the attitude towards climate change and policy, there might be an overarching latent motivational factor that drives response behavior across the whole item battery. The results of a principal-component factor analysis, displayed in Tab. 2, show that this is indeed the case. The analysis retains a single factor, which we call “motivation to contribute”, or “motivation” for short. This term is intuitive, as “moral duty” and “urgency” load strongly positively, and “hype” strongly negatively on the factor. “Public policy” also loads positively, but rather weakly. Using the Bartlett scoring coefficients, we can construct a “motivation” score for each participant that is used for deeper analysis of behavior in the experiment. The in-sample distribution of scores is bi-modal and highly skewed, with the absolute majority at the high end.

Supplementary Table 2. Results of a maximum likelihood principal-component factor analysis of four motivational aspects.

	Factor “Motivation”		
	Loading[a]	Uniqueness[b]	Scoring[c]
Moral duty	0.7264	0.4724	0.1762
Public policy	0.3483	0.8787	0.0454
Urgency	0.9308	0.1337	0.7981
Hype	−0.7052	0.5027	−0.1608
Eigenvalue	2.0125		
Observations	4,094[d]		
$\log \mathscr{L}$	−1.2982[e]		
Schwarz’s BIC	35.8655		
Akaike’s AIC	10.5963		

[a] Factor loadings
[b] Unique variances
[c] Bartlett scoring coefficients
[d] Non-response category “don’t know/prefer not to say” set to missing, which drops 45 out of 4,139 subjects.
[e] A likelihood ratio test independent vs. saturated yields $\chi^2(6) = 5341.08$, rejecting the null at $p = 0.0000$. A likelihood ratio test 1 factor vs. saturated yields $\chi^2(2) = 2.59$, not rejecting the null at conventional significance levels ($p = 0.2733$).

A.2 Knowledge of the EU Emissions Trading System

The final item of the pre-experimental battery is different in that it is not about motivation but a self-assessment of the individual knowledge about the EU ETS as an established climate policy instrument – the most important in the participants’ jurisdiction. This item asks “Could you explain to a friend how the EU Emissions Trading System works?”, with response categories being “I could explain the system in detail”, “I could explain the system roughly”, “I could not explain the system”, and “I don’t know the system”. The majority of 60.1 % of the participants does not know or understand the ETS (Tab. 3). Hence, for the majority of participants, the choice task involves significant uncertainty. In other words, there is enough scope for learning, which is important for our experiment.

Supplementary Table 3. Subjects’ knowledge of the EU ETS.

Item	could explain in detail	could roughly explain	could not explain	don’t know the system	n/a[b]	Total
ETS[a]	78	1,537	1,933	552	39	4,109
	1.9%	37.1%	46.7%	13.4%	0.9%	100.0%

[a] “Could you explain to a friend how the EU Emissions Trading System works?”
[b] Non-response category “don’t know/prefer not to say”

B Manipulation check

A simple manipulation check ascertains that (i) subjects spent a slightly longer period on the decision screen in the timing conditions ($z > 1$) compared to the no-timing condition ($z = 1$), and (ii) a successively increasing period on the advice and decision screens in the advice conditions $z = 3$ through $z = 5$ (Tab. 4). However, given the length of the introduction and advice screens (see Supplementary Information E), it can be assumed that respondents who spent less than the median time on the respective screens only paid little attention to them.

Supplementary Table 4. Median screening durations (in seconds) of the introduction, advice, and decision parts of the experimental instructions.

		Condition-specific screens		
Condition	Introduction[a]	Advice[b]	Decision[c]	Total
No timing ($z = 1$)	83		21	21
Simple timing & zero advice ($z = 2$)	76		35	35
Simple timing & minimal advice ($z = 3$)[d]	74		49	49
Simple timing & extensive advice ($z = 4$)	77	31	35	66
Sophisticated timing & extensive advice ($z = 5$)	74	30	40	70
χ^2-statistic[e]	4.7371	0.5408	450.0801	
p-value [e]	0.3154	0.4621	0.0000	

[a] Introductory screens shown identically to all subjects. Full text in Appendices E.1 and E.2.
[b] Condition-specific screens including the advice. Full text in Appendices E.3 through E.7, up to the sentence "How do you decide?", respectively.
[c] Condition-specific screens including the choice task, down from the sentence "How do you decide?", respectively.
[d] In $z = 3$ the advice was displayed on the decision screen.
[e] χ^2-statistics with ties and the p-values for a Kruskal-Wallis equality-of-populations rank test with 4 degrees of freedom for Introduction and Decision and one degree of freedom for Advice.

C Treatment Effect Heterogeneity by Motivated Reasoning and Need for Cognition

C.1 The Role of Motivated Reasoning

Motivated beliefs are difficult to update, as they tend to be more inert to conflicting information than "rational" beliefs[1–4]. Hence, the treatment effects could differ between those who agree with the pre-experimental survey items "moral duty" and "urgency" and those who disagree. The treatment effect estimates from extended probit estimations in which the four motivational items are included indicate, though, that this is only partly the case (Tab. 5). Generally, the treatment effects on WTC tend to be positive subject to disagreement with the "moral duty" statement, but negative subject to agreement, yet not all differences are statistically significant. With respect to the "urgency" item, there is no evidence for heterogeneous treatment effects on WTC.

Likewise, there is no heterogeneity in the treatment effects on REC with respect to the "moral duty" statement. However, the treatment effects are systematically bigger among those who agree with the "urgency" statement compared to those who disagree. This result contrasts with the motivated reasoning conjecture: Those who *ex ante* believe that climate action is urgent are more responsive to information that states that waiting increases effectiveness. Given this evidence, the role of motivated reasoning seems limited.

We know from the factor analysis presented in Supplementary Information A that the responses to the four motivational items are not independent: the analysis retained a single principal factor that can be interpreted as a general "motivation to contribute." Using the Bartlett scoring coefficients to construct a "motivation" score for each participant, we can use that score in the regressions, instead of the individual items. While not reporting the estimates here because the "motivation" scores have no natural metric over and above their ordinal ordering, such that effect sizes are difficult to interpret, it turns out that all interaction effects between the treatment indicators and the score are statistically insignificant for WTC, but highly significant and clearly positive for REC: the treatment effect sizes are increasing with the "motivation" score. In short, treatment effects are heterogeneous for REC, but not for WTC.

One concern is that the results reflect an experimenter demand effect. First, note that at no point the instruction alluded to which action should be taken, it is just the mere statement that one action is more effective than another which can be taken as a recommendation. However, the pull of an experimenter demand effect over and above the informational value of the statement should be independent of any attitude towards the issue at hand. Hence, given the heterogeneity in beliefs for urgent action, those who think the need for climate action is less pressing should delay retirement more often. Yet, we find the opposite. Moreover, the association of information on the effectiveness of a particular course of action with a recommendation to (not) pursue it would in most real-world settings be even more salient than in our experiment.

Supplementary Table 5. Moderating effects of the motivational pre-experimental item battery on WTC and REC.

	Willingness to contribute (WTC)[a]				Relative effectiveness of contribution (REC)[c]			
	Moral Duty		Urgency		Moral Duty		Urgency	
Reference margin[c]	0.6450	(0.0197)***	0.6292	(0.0217)***	0.5281	(0.0323)***	0.7085	(0.0304)***
Effect of agreement	0.1584	(0.0228)***	0.1863	(0.0248)***	0.0763	(0.0345)**	−0.1259	(0.0328)***
Treatment effects at disagreement:								
$z = 2$	0.0577	(0.0861)	0.0275	(0.0830)				
$z = 3$	0.1715	(0.0775)**	0.1045	(0.0791)	0.1851	(0.1044)*	0.0623	(0.0991)
$z = 4$	0.1015	(0.0789)	0.0460	(0.0810)	0.2796	(0.1040)***	0.0440	(0.0975)
$z = 5$	0.1997	(0.0751)***	−0.0261	(0.0802)	0.2621	(0.0993)***	0.1587	(0.0930)*
Treatment effects at agreement:								
$z = 2$	−0.0448	(0.0274)	−0.0397	(0.0293)				
$z = 3$	−0.0307	(0.0265)	−0.0188	(0.0283)	0.2582	(0.0296)***	0.2743	(0.0306)***
$z = 4$	−0.0284	(0.0259)	−0.0174	(0.0284)	0.3107	(0.0285)***	0.3403	(0.0298)***
$z = 5$	−0.0088	(0.0256)	0.0466	(0.0264)*	0.3400	(0.0285)***	0.3553	(0.0297)***

Estimates derived from a maximum likelihood probit regression with four full factorial interactions of z and the pre-experimental item variables moral duty, urgency, public policy, and hype (in reduced coding, coding neither nor/disagreement/full disagreement as disagreement (= reference category) and agreement/full agreement as agreement). In parentheses are the bootstrap standard errors derived from 1,000 replications. Stars indicate that a Wald test rejects the null that the respective margin is uniform or the respective effect is equal to zero at conventional significance levels (* at $p < 0.1$, ** at $p < 0.05$, and *** at $p < 0.01$).

[a] $n = 4,094$, $\log \mathscr{L} = -1,790.81$, Wald $\chi^2(24) = 778.76$, Wald $p = 0.0000$, pseudo $R^2 = 0.1859$ for WTC.

[b] $n = 2,774$, $\log \mathscr{L} = -1,757.19$, Wald $\chi^2(19) = 213.79$, Wald $p = 0.0000$, pseudo $R^2 = 0.0607$ for REC.

[c] Reference margin is WTC or REC when having expressed a neutral attitude towards the respective pre-experimental item. "'Agreement effect" is the average partial effect of respective discrete changes from the reference category to agreement to the respective pre-experimental item statement. The four rows below "At Disagreement" list the treatment effects of respective discrete changes of z relative to the reference category subject to disagreement to the respective pre-experimental item statement. Likewise for the four rows below "At Agreement". Heterogeneous treatment effects would imply that the effects "At Disagreement" and "At Agreement" are significantly different from one another.

C.2 The Role of Need for Cognition

Another possible source of treatment effect heterogeneity is need for cognition. Understanding the explanation requires some careful reading and abstract thinking. People generally tend to avoid cognitive demand[5], but individuals differ with respect to their need for cognition, i.e. their "tendency to engage in and enjoy effortful cognitive endeavors" [6, p. 306].[7] show that need for cognition affects how individuals process persuasive messages. Moreover, need for cognition is likely to be positively correlated with cognitive ability. For our experiment, this means that those with higher cognitive skill (and hence lower marginal cost of engaging with the explanation) are more likely to read and process the explanation.

We measured need for cognition post-experimentally by means of the standard Need for Cognition (NFC) test. The test consists of four statements to which the respondent can express applicability on a seven-point Likert scale (1 = does not apply at all, 7 = does fully apply): (i) "It is enough for me simply to know the answer without understanding the reasons for the answer of a problem," (ii) "I like my life to be full of tricky tasks to solve," (iii) "I would prefer more complicated problems to simple problems,", and (iv) "First and foremost, I think because I have to.". From the four items we constructed a simple need for cognition score for each participant by summing up the response codes (appropriately inverting the scales for the first and last item). Thus, the minimum achievable score is 4 and the maximum is 28. The distribution of the score in the sample is unimodal (mode = 16) with a mean of 17.8 (s. d. 3.9) and a median of 18.

Classifying individuals with a score at or below the mid-point as having a low need for cognition and those with a score above the mid-point as having a high need for cognition (we opt for this conservative classification because we have no reason to assume that the NFC measure is actually interval-scaled), we can check for treatment effect heterogeneity in probit regressions with interaction effects between the treatment indicators and the need for cognition indicator. It turns out that the treatment effects are not significantly different for individuals with high and low need for cognition for both WTC and REC. Hence, there is no evidence of treatment effect heterogeneity with respect to need for cognition. There is, however, a highly significant independent effect of need for cognition on the WTC: on average, switching from the low to the high need for cognition class increases the probability of EUA retirement by seven percentage points.

Supplementary Table 6. Moderating effects of need for cognition (NFC) on WTC and REC.

	WTC		REC	
Reference margin	0.7365	(0.0115)***	0.6026	(0.0155)***
Effect of high NFC	0.0693	(0.0139)***	−0.0017	(0.0194)
Treatment effects at low NFC:				
$z = 2$	−0.0843	(0.0427)**		
$z = 3$	−0.0032	(0.0379)	0.1688	(0.0501)***
$z = 4$	−0.0855	(0.0408)**	0.2541	(0.0502)***
$z = 5$	0.0093	(0.0399)	0.3040	(0.0489)***
Treatment effects at high NFC:				
$z = 2$	−0.0372	(0.0308)		
$z = 3$	−0.0025	(0.0296)	0.2734	(0.0274)***
$z = 4$	−0.0034	(0.0284)	0.3259	(0.0374)***
$z = 5$	−0.0108	(0.0287)	0.3350	(0.0355)***
Observations	3,764		2,570	
$\log \mathscr{L}$	−1,967.00		−1,659.04	
Wald χ^2	41.72		125.01	
Wald p	0.0000		0.0000	
pseudo R^2	0.0100		0.0404	

Estimates derived from a maximum likelihood probit regression. In parentheses are the bootstrap standard errors derived from 1,000 replications. Stars indicate that a Wald test rejects the null that the respective margin is uniform or the respective effect is equal to zero at conventional significance levels (* at $p < 0.1$, ** at $p < 0.05$, and *** at $p < 0.01$).
Reference margin is WTC or REC at low NFC. "Effect of high NFC" is the average partial effect of a discrete change from low to high NFC.
Heterogeneous treatment effects would imply that treatment effects at low NFC and at high NFC are significantly different from one another.

D Tables

Supplementary Table 7. Planned and sampled count of subjects by experimental condition, and the respective count of completes.

Condition	Planned	Sampled	Completed[a]
No timing ($z = 1$)	400	513	491
Simple timing & zero advice ($z = 2$)	600	704	665
Simple timing & minimal advice ($z = 3$)	1,000	1,073	996
Simple timing & extensive advice ($z = 4$)	1,000	1,076	989
Sophisticated timing & extensive advice ($z = 5$)	1,000	1,078	998
Total	4,000	4,444	4,139

[a] Sampled count of subjects net of dropouts. Since participation in the survey was voluntary, subjects could refuse to participate or drop out at any point of the survey.

Supplementary Table 8. Summary statistics by experimental condition

	z = 1	z = 2	z = 3	z = 4	z = 5	χ^{2a}	p-value[a]
Male	0.552	0.582	0.580	0.555	0.525	8.057	0.0895
Age	56.00	56.22	55.44	54.80	56.01	3.932	0.4152
Qual. for university entrance	0.509	0.513	0.542	0.524	0.517	2.276	0.6852
Employed	0.528	0.499	0.517	0.534	0.524	2.065	0.7237
Income < 1,200 Euro	0.056	0.054	0.071	0.067	0.057	2.840	0.5850
Income 1,200 - 2,700 Euro	0.296	0.361	0.304	0.282	0.317	10.866	0.0281
Income 2,700 - 4,200 Euro	0.313	0.287	0.337	0.356	0.310	9.192	0.0565
Income $\geq$ 4,200	0.336	0.298	0.288	0.296	0.316	4.021	0.4032
Household size:							
1 person	0.228	0.264	0.262	0.258	0.275	3.860	0.4253
2 persons	0.475	0.498	0.464	0.473	0.452	3.603	0.4624
3 persons	0.132	0.109	0.143	0.121	0.140	5.700	0.2227
4 and more persons	0.165	0.130	0.132	0.148	0.134	4.585	0.3326

[a] χ^2-statistics with ties and the p-values for a Kruskal-Wallis equality-of-populations rank test with 4 degrees of freedom.

Supplementary Table 9. Comparison of the Sample with the German Population

	Sample	Population
Male	55.8%	49.5%
Qualification for university entrance	52.3%	30.8%
Employed	52.1%	50.5%
High income	30.4%	24.8%
Age < 25 years	2.3%	24.2%
Age 25 - 64 years	63.8%	54.4%
Age $\geq$ 65 years	34.0%	21.4%
Household size:		
1 person	26.0%	20.7%
2 persons	47.0%	33.3%
3 persons	13.0%	17.7%
4 and more persons	14.0%	28.3%

Data for the German population in 2021 is taken from[8]. In that survey, the threshold for high income is €4,000, whereas we set it at €4,200.

Supplementary Table 10. Average treatment effects on WTC, estimated via maximum likelihood probit regression

	Null Model		Partial Model		Full Model	
$z = 1$ margin	0.7862	$(0.0186)^{***}$	0.7862	$(0.0187)^{***}$	0.7862	$(0.0188)^{***}$
$z = 2$ vs. $z = 1$ effect			−0.0553	$(0.0247)^{**}$	−0.0553	$(0.0258)^{**}$
$z = 3$ vs. $z = 1$ effect					−0.0100	(0.0231)
$z = 4$ vs. $z = 1$ effect					−0.0319	(0.0228)
$z = 5$ vs. $z = 1$ effect					0.0004	(0.0229)
Observations	491		1,156		4,139	
$\log \mathscr{L}$	-254.84		-642.15		-2,240.68	
Wald χ^2			4.89		9.01	
Wald p			0.0270		0.0608	
Pseudo R^2	0.0000		0.0037		0.0021	

Predictive margins for $z = 1$ and average effects of the respective discrete change of z relative to $z = 1$. In parentheses are the bootstrap standard errors derived from 1,000 replications. Stars indicate that a Wald test rejects the null that the respective margin is uniform or the respective effect is equal to zero at conventional significance levels (* at $p < 0.1$, ** at $p < 0.05$, and *** at $p < 0.01$).

Supplementary Table 11. Average treatment effects on WTC relative to $z = 2$, estimated via maximum likelihood probit regression.

	Full Model	
$z = 2$ margin	0.7308	$(0.0174)^{***}$
$z = 3$ vs. $z = 2$ effect	0.0453	$(0.02181)**$
$z = 4$ vs. $z = 2$ effect	0.0235	(0.0220)
$z = 5$ vs. $z = 2$ effect	0.0557	$(0.0214)^{***}$
Observations	3,648	
$\log \mathscr{L}$	−1,985.84	
Wald χ^2	8.12	
Wald p	0.0436	
Pseudo R^2	0.0020	

Predictive margins for $z = 2$ and average effects of the respective discrete change of z relative to $z = 2$, derived from a maximum likelihood probit regression. In parentheses are the bootstrap standard errors derived from 1,000 replications. Stars indicate that a Wald test rejects the null that the respective margin is uniform or the respective effect is equal to zero at conventional significance levels (* at $p < 0.1$, ** at $p < 0.05$, and *** at $p < 0.01$).

Supplementary Table 12. Average treatment effects on WTC, estimated via maximum likelihood probit regression, with covariates.

	Full Model with covariates	
$z = 1$ margin	0.8056	$(0.0194)^{***}$
$z = 2$ vs. $z = 1$ effect	−0.0520	$(0.0262)^{**}$
$z = 3$ vs. $z = 1$ effect	−0.0068	(0.0238)
$z = 4$ vs. $z = 1$ effect	−0.0369	(0.0247)
$z = 5$ vs. $z = 1$ effect	−0.0142	(0.0240)
Male	−0.0778	$(0.137)^{***}$
Income $1,200 - 2,700$ €	0.0531	$(0.0321)^{*}$
Income $2,700 - 4,200$ €	0.0744	$(0.0325)^{**}$
Income $\geq 4,200$ €	0.1223	$(0.0323)^{***}$
Observations	3,609	
$\log \mathscr{L}$	−1,856.47	
Wald χ^2	57.46	
Wald p	0.0000	
Pseudo R^2	0.0158	

Predictive margins for $z = 1$ and average effects of the respective discrete change of z relative to $z = 1$, derived from a maximum likelihood probit regression. In parentheses are the bootstrap standard errors derived from 1,000 replications. Stars indicate that a Wald test rejects the null that the respective margin is uniform or the respective effect is equal to zero at conventional significance levels (* at $p < 0.1$, ** at $p < 0.05$, and *** at $p < 0.01$).

Supplementary Table 13. Average treatment effects on WTC, estimated via maximum likelihood probit regression, when including subjects that dropped out during the experiment.

	Null Model		Partial Model		Full Model	
$z = 1$ margin	0.7751	$(0.0180)^{***}$	0.7751	$(0.0186)^{***}$	0.7751	$(0.0190)^{***}$
$z = 2$ vs. $z = 1$ effect			-0.0625	$(0.0251)^{**}$	-0.0625	$(0.0253)^{**}$
$z = 3$ vs. $z = 1$ effect					-0.0290	(0.0232)
$z = 4$ vs. $z = 1$ effect					-0.0543	$(0.0237)^{**}$
$z = 5$ vs. $z = 1$ effect					-0.0253	(0.0237)
# of observations	498		1,180		4,298	
$\log \mathscr{L}$	-265.5		-674.5		-2,463.4	
Wald χ^2			6.0		8.5	
Wald p			0.0142		0.0759	
Pseudo R^2	0.0000		0.0043		0.0017	

Predictive margins for $z = 1$ and average effects of the respective discrete change of z relative to $z = 1$. In parentheses are the bootstrap standard errors derived from 1,000 replications. Stars indicate that a Wald test rejects the null that the respective margin is uniform or the respective effect is equal to zero at conventional significance levels (* at $p < 0.1$, ** at $p < 0.05$, and *** at $p < 0.01$).

Supplementary Table 14. Average treatment effects on REC, estimated via maximum likelihood probit regression

	Null Model		Full Model		With Covariates	
$z = 2$ margin	0.3560	$(0.0218)^{***}$	0.3560	$(0.0220)^{***}$	0.3517	$(0.0233)^{***}$
$z = 3$ vs. $z = 2$ effect			0.2430	$(0.0291)^{***}$	0.2575	$(0.0293)^{***}$
$z = 4$ vs. $z = 2$ effect			0.3009	$(0.0283)^{***}$	0.3025	$(0.0285)^{***}$
$z = 5$ vs. $z = 2$ effect			0.3256	$(0.0278)^{***}$	0.3239	$(0.0296)^{***}$
Covariates:	No		No		Gender, income	
Observations	486		2,790		2,478	
$\log \mathscr{L}$	-316.4		-1,807,9		-1,601.0	
Wald χ^2			142.9		144.3	
Wald p			0.0000		0.0000	
Pseudo R^2	0.0000		0.0398		0.0427	

Predictive margins for $z = 2$ and average effects of the respective discrete change of z relative to $z = 2$. In parentheses are the bootstrap standard errors derived from 1,000 replications. Stars indicate that a Wald test rejects the null that the respective margin is uniform or the respective effect is equal to zero at conventional significance levels (* at $p < 0.1$, ** at $p < 0.05$, and *** at $p < 0.01$).

Supplementary Table 15. Average treatment effects on REC relative to $z = 3$, estimated via maximum likelihood probit regression

	Full Model	
$z = 3$ margin	0.5990	$(0.0179)^{***}$
$z = 4$ vs. $z = 3$ effect	0.0579	$(0.0257)^{**}$
$z = 5$ vs. $z = 3$ effect	0.0826	$(0.0245)^{***}$
Observations	$2,304$	
$\log \mathscr{L}$	$-1,491.51$	
Wald χ^2	12.18	
Wald p	0.0023	
Pseudo R^2	0.0041	

Predictive margins for $z = 3$ and average effects of the respective discrete change of z relative to $z = 3$. In parentheses are the bootstrap standard errors derived from 1,000 replications. Stars indicate that a Wald test rejects the null that the respective margin is uniform or the respective effect is equal to zero at conventional significance levels (* at $p < 0.1$, ** at $p < 0.05$, and *** at $p < 0.01$).

Supplementary Table 16. Moderating effects of attention paid to explanations on introduction and advice screens on WTC and REC.

	WTC		REC	
Reference margin	0.7083	(0.0100)***	0.4775	(0.0142)***
Effect of attention on explanations	0.1170	(0.0130)	0.2162	(0.0183)***
Treatment effects without attention to explanations:				
$z = 2$	−0.0567	(0.0016)		
$z = 3$	0.0016	(0.0343)	0.1095	(0.0430)**
$z = 4$	−0.0694	(0.0360)*	0.0936	(0.0432)**
$z = 5$	−0.0045	(0.0349)	0.1857	(0.0419)***
Treatment effects with attention to explanations:				
$z = 2$	−0.0560	(0.0346)		
$z = 3$	−0.0212	(0.0299)	0.3620	(0.0375)***
$z = 4$	0.0057	(0.0283)	0.4636	(0.0359)***
$z = 5$	0.0065	(0.0282)	0.4437	(0.0361)***
Observations	4,135		2,788	
$\log \mathscr{L}$	−2,194.82		−1,712.13	
Wald χ^2	96.92		322.96	
Wald p	0.0000		0.0000	
pseudo R^2	0.0215		0.900	

Estimates derived from a maximum likelihood probit regression. In parentheses are the bootstrap standard errors derived from 1,000 replications. Stars indicate that a Wald test rejects the null that the respective margin is uniform or the respective effect is equal to zero at conventional significance levels (* at $p < 0.1$, ** at $p < 0.05$, and *** at $p < 0.01$).
Reference margin is WTC or REC without paying attention to explanations. "Effect of attention on explanations" is the average partial effect of a discrete change from no attention to attention.
Heterogeneous treatment effects would imply that treatment effects without attention and with attention are significantly different from one another.

Supplementary Table 17. Moderating effects of attention paid to introduction screens on WTC and REC.

	WTC		REC	
Reference margin	0.7118	(0.0099)***	0.4978	(0.0141)***
Effect of attention on introduction	0.1116	(0.0128)***	0.1848	(0.0185)***
Treatment effects without attention to introduction:				
$z = 2$	−0.0453	(0.0392)		
$z = 3$	0.0090	(0.0357)	0.1123	(0.0428)***
$z = 4$	−0.0403	(0.0376)	0.1179	(0.0432)***
$z = 5$	0.0108	(0.0358)	0.2257	(0.0413)***
Treatment effects with attention to introduction:				
$z = 2$	−0.0533	(0.0336)		
$z = 3$	−0.0149	(0.0287)	0.3673	(0.0378)***
$z = 4$	−0.0131	(0.0274)	0.4504	(0.0361)***
$z = 5$	0.0092	(0.0274)	0.4202	(0.0375)***
Observations	4,135		2,788	
$\log \mathscr{L}$	−2,201.13		−1,735.39	
Wald χ^2	85.05		284.21	
Wald p	0.0000		0.0000	
pseudo R^2	0.0187		0.0776	

Estimates derived from a maximum likelihood probit regression. In parentheses are the bootstrap standard errors derived from 1,000 replications. Stars indicate that a Wald test rejects the null that the respective margin is uniform or the respective effect is equal to zero at conventional significance levels (* at $p < 0.1$, ** at $p < 0.05$, and *** at $p < 0.01$).
Reference margin is WTC or REC without paying attention to the introduction. "Effect of attention on introduction" is the average partial effect of a discrete change from no attention to attention.
Heterogeneous treatment effects would imply that treatment effects without attention and with attention are significantly different from one another.

E Transscript of Experimental Instructions

Note that the bold-face text was not highlighted in the survey questionnaire, but is highlighted here to indicate the difference between conditions.

E.1 General Introduction for All Participants

In the following, you can claim a bonus of 5 Euros in the form of an Amazon voucher. However, you can also decide to forego this bonus in order to personally make an active contribution to climate protection by preventing the emission of one ton of carbon dioxide (CO_2). To put this into perspective, one metric ton is the average amount emitted by each German (through consumption, electricity use, heating and mobility) over the course of five weeks.
How exactly one can prevent the emission of this ton of CO_2 and what your decision situation looks like is explained on the following pages. You make your decision afterwards.

Regardless of whether you decide to receive the 5-Euro bonus in the form of an Amazon voucher or to make an active contribution to climate protection, we will only implement the decision of every fourth participant. To do this, we will randomly select 1,000 participants from the total of 4,000 participants after the survey has ended. If you are one of them, you will receive the 5-Euro-bonus or we will implement the active contribution to climate protection—depending on how you have decided.

E.2 Specific Introduction for All Participants

The European Union (EU) wants to quickly and significantly reduce emissions of climate-damaging greenhouse gases (e.g., carbon dioxide, CO_2) and achieve climate neutrality by 2050. In order to effectively control the emission of these pollutants, large power plants and industrial facilities must acquire and surrender a so-called emission allowance for each ton of greenhouse gas they emit. The emission allowance is then used up forever and cannot be used again. The amount of these emission allowances issued by the EU is strictly limited.
You are now given the opportunity to decide whether an emission allowance is irreversibly withdrawn from circulation. This is called "retiring". A retired emission allowance is no longer available to power plants. The power plants can therefore emit one ton of CO_2 less. This effectively reduces overall emissions in the EU and makes an active contribution to climate protection.
A confirmation of retirement will be sent to you by e-mail after completion of the survey if you have decided to retire an emission allowance and if you are one of the participants whose decision will be implemented.

E.3 Choice Setting in Condition $z = 1$

You now can retire an emission allowance that entitles you to emit one ton of CO_2. This will not incur any costs for you. However, you will forgo your bonus of 5 Euros.

How do you decide?

- Climate protection: I waive my 5-Euro bonus and want an emission allowance to be retired on a binding basis.
- Consumption: I want to keep my 5-Euro bonus. No emission allowance will be retired.
- don't know/no answer

E.4 Choice Setting in Condition $z = 2$

You now can retire an emission allowance that entitles to emit one ton of CO_2. **You can decide whether the retirement occurs either immediately or in one year. Even if you decide to retire in one year, this decision is binding and cannot be reversed later.** This will not incur any costs for you. However, you will forgo your bonus of 5 Euros.

How do you decide?

- Climate protection variant A: I waive my 5-Euro bonus and want an emission allowance to be retired immediately.
- Climate protection variant B: I waive my 5-Euro bonus and want an emission allowance to be retired in a year's time.
- Consumption: I want to keep my 5-Euro bonus. No emission allowance will be retired.
- don't know/no answer

E.5 Choice Setting in Condition $z = 3$

You now can retire an emission allowance that entitles to emit one ton of CO_2. You can decide whether the retirement occurs either immediately or in one year. Even if you decide that retirement occurs in one year, this decision is binding and cannot be reversed later. This will not incur any costs for you. However, you will forgo your bonus of 5 Euros.

The timing of the retirement makes a difference. The number of emission allowances issued next year depends on the number of emission allowances retired this year. Therefore, retiring now (variant A) reduces total emissions by 0.24 tons of CO_2 less than retiring in one year (variant B). Retiring in one year (variant B) is the greater contribution to climate protection.

How do you decide?

- Climate protection variant A: I waive my 5-Euro bonus and want an emission allowance to be retired immediately.
- Climate protection variant B: I waive my 5-Euro bonus and want an emission allowance to be retired in a year's time.
- Consumption: I want to keep my 5-Euro bonus. No emission allowance will be retired.
- don't know/no answer

E.6 Choice Setting in Condition $z = 4$

You now can retire an emission allowance that entitles you to emit one ton of CO_2. You can decide whether the retirement occurs either immediately or in one year. Even if you decide that retirement occurs in one year, this decision is binding and cannot be reversed later. This will not incur any costs for you. However, you will forgo your bonus of 5 Euros.

The timing of retirement makes a difference due to the Market Stability Reserve. It reduces the number of emission allowances available in the future based on the number of emission allowances that are not used at the end of a calendar year but are carried over by the power plant operators into the next year. If an emission allowance is now purchased and immediately retired, the number of emission allowances carried over at the end of a year is reduced.

This in turn means that more emission allowances are issued than would have been the case without the retirement. Although retirement deprives power plants of an emission allowance, this is partially (24% per year) offset by the Market Stability Reserve in the future. However, if a purchased emission allowance is not retired immediately, but is set aside and only retired later, then the emission allowance is still counted as part of the carryover at the end of a calendar year.

The allowance therefore continues to trigger a reduction in the number of allowances issued in the future via the Market Stability Reserve. Power plant operators are therefore not only deprived of the emission allowance purchased and later retired, but fewer new ones are made available.

In summary, this means: The timing of the retirement makes a difference. The number of emission allowances issued next year depends on the number of emission allowances retired this year. Therefore, retiring now (variant A) reduces total emissions by 0.24 tons of CO_2 less than retiring in one year (variant B). Retiring in one year (variant B) is the greater contribution to climate protection.

How do you decide?

- Climate protection variant A: I waive my 5-Euro bonus and want an emission allowance to be retired immediately.
- Climate protection variant B: I waive my 5-Euro bonus and want an emission allowance to be retired in a year's time.
- Consumption: I want to keep my 5-Euro bonus. No emission allowance will be retired.
- don't know/no answer

E.7 Choice Setting in Condition $z = 5$

You can now retire an emission right that entitles you to emit one ton of CO_2. You can decide whether the retirement occurs either immediately or at a future date. Even if you decide that retirement occurs at a future date, this decision is binding and cannot be reversed later. This will not incur any costs for you. However, you will forgo your bonus of 5 Euros.

The timing of retirement makes a difference due to the Market Stability Reserve. It reduces the number of emission rights available in the future based on the number of emission rights that are not used at the end of a calendar year but are carried over by the power plant operators into the next year. If an emission right is now purchased and immediately retired, the number of emission rights carried over at the end of a year is reduced.

This in turn means that more emission rights are issued than would have been the case without the retirement. Although retirement deprives power plants of an emissions allowance, this is partially (24% per year until 2023, 12% per year thereafter) offset by the Market Stability Reserve in the future. **This is true for each year that the Market Stability Reserve is active. This is expected to be the case for several more years.** However, if a purchased emission allowance is not retired immediately, but is set aside and only retired later, then the emission allowance is still counted as part of the carryover at the end of each calendar year.

The allowance therefore continues to trigger a reduction in the number of allowances issued in the future via the Market Stability Reserve. Power plant operators are therefore not only deprived of the emission allowance purchased and later retired, but fewer new ones are also made available each year.

In summary, this means: The timing of the retirement makes a difference. The number of emission allowances issued next year depends on the number of emission allowances retired this year. Therefore, retiring now (Option A) reduces total emissions by at least 0.24 tons of CO_2 less than retiring at a future date (Option B). However, it is likely that the difference in effectiveness between Variant A and Variant B is significantly higher. Retiring at a future date (variant B) is the greater contribution to climate protection.

How do you decide?

- Climate protection Option A: I waive my 5-euro bonus and want an emission right to be retired immediately.
- Climate protection option B: I waive my 5-euro bonus and want an emission allowance to be retired on a binding basis at an as yet undetermined future date when the climate impact of the retirement is greatest.
- Consumption: I want to keep my 5-euro bonus. No emission allowance will be retired.
- don't know/no answer

References

1. Eil, D. & Rao, J. M. The good news-bad news effect: Asymmetric processing of objective information about yourself. *Am. Econ. Journal: Microeconomics* **3**, 114–138 (2011).
2. Gershman, S. J. How to never be wrong. *Psychon. Bull. & Rev.* **26**, 13–28 (2019).
3. Kuzmanovic, B., Rigoux, L. & Tittgemeyer, M. Influence of vmPFC on dmPFC predicts valence-guided belief formation. *J. Neurosci.* **38**, 7996–8010 (2018).
4. Yao, Z., Lin, X. & Hu, X. Optimistic amnesia: How online and offline processing shape belief updating and memory biases in immediate and long-term optimism biases. *Soc. Cogn. Affect. Neurosci.* **16**, 453–462 (2021).
5. Kool, W., McGuire, J., Rosen, T. & Botvinik, M. M. Decision making and the avoidance of cognitive demand. *J. Exp. Psychol. Gen.* **139**, 665–682 (2010).

6. Cacioppo, J. T., Petty, R. E. & Feng Kao, C. The efficient assessment of need for cognition. *J. Pers. Assess.* **48**, 306–307 (1984).
7. Cacioppo, J. T., Petty, R. E. & Morris, K. J. Effects of need for cognition on message evaluation, recall, and persuasion. *J. Pers. Soc. Psychol.* **45**, 805 (1983).
8. Statistisches Bundesamt. Statistik 12211 (2022). https://www-genesis.destatis.de/genesis/online?sequenz=statistikTabellen&selectionname=12211#abreadcrumb.
